# Cell Type-Specific Differentiation Between *Indica* and *Japonica* Rice Root Tip Responses to Different Environments Based on Single-Cell RNA Sequencing

**DOI:** 10.3389/fgene.2021.659500

**Published:** 2021-05-17

**Authors:** Zhe Wang, Daofu Cheng, Chengang Fan, Cong Zhang, Chao Zhang, Zhongmin Liu

**Affiliations:** ^1^Shanghai Key Laboratory of Signaling and Disease Research, Translational Medical Center for Stem Cell Therapy and Institute for Regenerative Medicine, Frontier Science Center for Stem Cell Research, School of Life Sciences and Technology, Shanghai East Hospital, Tongji University, Shanghai, China; ^2^Department of Cardiac Surgery, School of Medicine, Shanghai East Hospital, Tongji University, Shanghai, China

**Keywords:** adaptative evolution, *indica-japonica* differentiation, *Oryza sativa*, quiescent center cells, single-cell RNA sequencing, transcription factor family

## Abstract

**Background:** As *Oryza sativa* ssp. *indica* and *Oryza sativa* ssp. *japonica* are the two major subspecies of Asian cultivated rice, the adaptative evolution of these varieties in divergent environments is an important topic in both theoretical and practical studies. However, the cell type-specific differentiation between *indica* and *japonica* rice varieties in response to divergent habitat environments, which facilitates an understanding of the genetic basis underlying differentiation and environmental adaptation between rice subspecies at the cellular level, is little known.

**Methods:** We analyzed a published single-cell RNA sequencing dataset to explore the differentially expressed genes between *indica* and *japonica* rice varieties in each cell type. To estimate the relationship between cell type-specific differentiation and environmental adaptation, we focused on genes in the WRKY, NAC, and BZIP transcription factor families, which are closely related to abiotic stress responses. In addition, we integrated five bulk RNA sequencing datasets obtained under conditions of abiotic stress, including cold, drought and salinity, in this study. Furthermore, we analyzed quiescent center cells in rice root tips based on orthologous markers in *Arabidopsis*.

**Results:** We found differentially expressed genes between *indica* and *japonica* rice varieties with cell type-specific patterns, which were enriched in the pathways related to root development and stress reposes. Some of these genes were members of the WRKY, NAC, and BZIP transcription factor families and were differentially expressed under cold, drought or salinity stress. In addition, *LOC*_*Os01g16810*, *LOC*_*Os01g18670*, *LOC*_*Os04g52960*, and *LOC*_*Os08g09350* may be potential markers of quiescent center cells in rice root tips.

**Conclusion:** These results identified cell type-specific differentially expressed genes between *indica*-*japonica* rice varieties that were related to various environmental stresses and provided putative markers of quiescent center cells. This study provides new clues for understanding the development and physiology of plants during the process of adaptative divergence, in addition to identifying potential target genes for the improvement of stress tolerance in rice breeding applications.

## Introduction

Asian cultivated rice (*Oryza sativa L*.) is one of the most important cereal crops and has been domesticated for approximately 7,000–8,000 years (Zong et al., 2007). As two geographically and genetically diverged subspecies, *Oryza sativa* ssp. *japonica* varieties are mainly grown in temperate regions at high latitudes, while *Oryza sativa* ssp. *indica* varieties are mostly cultivated in subtropical and tropical regions with low latitudes or altitudes (Liu et al., 2018; Campbell et al., 2020). As a result, cold, drought and salinity stresses are major limiting factors for rice survival and production in separate habitats (Gomez et al., 2010; Zhang et al., 2014; Liu et al., 2018). The differentiation of *indica* and *japonica* rice reflects the adaptative evolution of plant species in divergent natural and human-influenced environments, in addition to that through direct artificial selection (Gross and Olsen, 2010). The key to rice subspecific differentiation is natural genetic variations that involve environmental adaptation and human preferences (Ma et al., 2015; Liu et al., 2019; Xu et al., 2020). For example, *COLD1^*jap*^* and *bZIP73^*jap*^* were selected as natural variants during the process of *japonica* domestication, which can partially explain the cold tolerance of *japonica* rice varieties (Ma et al., 2015; Liu et al., 2019). *SLG1^*indica*^* contributed to adaptation to high-temperature environments through artificial selection in *indica* domestication (Xu et al., 2020). In addition to the genetic variation between *indica* and *japonica*, the level of gene expression is also very important in adaptation to divergent environments (Campbell et al., 2020).

Transcription factors play an important role in the regulation of gene expression and related biological processes that result in changes in plant biochemistry, physiology, and morphology (Nuruzzaman et al., 2010; Liu et al., 2018; Nan et al., 2020). Previous studies showed that many genes in the BZIP, NAC, and WRKY transcription factor families can participate in responses to various biotic and abiotic stresses, such as cold, salt, and drought (Nijhawan et al., 2008; Zheng et al., 2009; Nuruzzaman et al., 2010; Manu et al., 2017; Liu et al., 2018; Nan et al., 2020). For example, Liu et al. (2018) found that *bZIP73* in BZIP transcription factor families could significantly enhance the cold tolerance ability in rice, suggesting a potential contribution to the northward expansion of the *japonica* rice variety. In addition, *bZIP73* can be a promising marker of enhanced cold tolerance in rice breeding, according to Liu et al. (2018). Therefore, the study of the BZIP, NAC, and WRKY transcription factor families, which are closely related to abiotic stress responses, has an important role in understanding the genetic basis of differentiation and environmental adaptation between rice subspecies in addition to the selection of tolerant genes in rice breeding applications.

Commonly, there are a variety of cell types in multicellular plants, including cultivated rice, and these cells work as an interactive network to maintain the survival and reproduction of individual plants (Liu et al., 2020; Wang et al., 2020) in addition to responding to stresses and adapting to various environments. Different organs and even different cell types have their own roles in the regulation of organisms—for example, the cortex is related to water and fluid transport, and the epidermis near root hairs is related to extracellular and external stimuli in rice roots (Liu et al., 2020). Therefore, understanding the differences in gene expression among different organs and different cell types is important for understanding the development and physiology of plants (Denyer et al., 2019; Ryu et al., 2019; Shulse et al., 2019; Liu et al., 2020). However, analyses involving traditional bulk RNA sequencing cannot identify cell type-specific heterogeneity due to the mixture of cell types in the test samples (Dai et al., 2019). In the last decade, the development of single-cell RNA sequencing has provided a new opportunity to study the heterogeneity of cell types in plants (Denyer et al., 2019; Ryu et al., 2019; Shulse et al., 2019; Liu et al., 2020). In addition, quiescent center (QC) cells are an important group of cells in plant root tips that play a key role in the control of tip growth and the regulation of stem cells in roots (Ryu et al., 2019). However, the understanding of QC cells is very limited due to the lack of cell-specific gene expression tools for these cells, which is an issue that could be solved by single-cell RNA sequencing.

In this study, we reanalyzed a published single-cell RNA sequencing dataset to explore the cell type-specific differentially expressed genes (DEGs) between *indica* and *japonica* rice varieties. To estimate the relationship between cell type-specific differentiation and environmental adaptation of *indica* and *japonica* rice varieties, we also analyzed five bulk RNA sequencing datasets under abiotic stresses, including cold, drought and salinity, and focused on stress-induced DEGs in the WRKY, NAC, and BZIP transcription factor families, which are closely related to abiotic stress responses. In addition, we also used orthologous markers of QC cells from *Arabidopsis* to identify QC cells and putative QC markers in rice root tips. The objectives of this study were (1) to identify the cell type-specific differentiation between *indica* and *japonica* rice varieties in root tip responses to environmental adaptation and (2) to provide potential markers of QC cells or cells in the initial stage of root development that were relatively close to QC cells. This study examined the cell type-specific differentiation between *indica* and *japonica* rice varieties during the process of adaptation to various environments, which could provide new clues regarding the development and physiology of plants during adaptative evolution, in addition to having applications in breeding.

## Materials and Methods

### Identification of Cell Types in *Indica* or *Japonica* Rice Root Tips Based on Single-Cell RNA Sequencing

The raw count matrix data of *Oryza sativa* ssp. *indica* variety 93-11 and *Oryza sativa* ssp. *japonica* variety Nipponbare from a previously published paper (GSE146035) (Liu et al., 2020), which included more than 20,000 single cells from 250 tips of crown roots, were used in this study. Cells with fewer than 1000 detected genes and genes detected in fewer than 10 cells were removed from further analysis. After scaling and normalization, the top 2000 highly variable genes were used for principal component analysis (PCA) dimensionality reduction. The first 50 principal components (PCs) with a resolution parameter of 1.0 were used for clustering, and UMAP (Becht et al., 2018) was used to visualize clusters. We used the gene markers of eight previously reported cell types (Liu et al., 2020) to identify cell types in *indica* or *japonica* rice varieties. Then, we combined the cells from *indica* or *japonica* rice varieties with the removal of batch effects. DEGs between *indica* and *japonica* rice varieties (defined as *indica*-*japonica* DEGs) in each cell type were identified using the function FindAllMarkers with an aver_logFC > 0.25 and *P* < 0.01. All analyses above were performed using Seurat package V3.0.2 (Butler et al., 2018; Stuart et al., 2019) in R. Shared genes between different cell types were calculated by FunRich_2.1.2 software (Pathan et al., 2015). Both KEGG (Kyoto Encyclopedia of Genes and Genomes) and GO (Gene Ontology) enrichment analyses were conducted using PlantGSEA^1^. The top 15 enriched KEGG and GO terms (BP, Biological Process; CC, Cellular Component; MF, Molecular Function) of DEGs were visualized using the ggplot2 package V3.2.1 (Wickham, 2016) in R.

### Identification of Differentially Expressed Cell Type-Specific Genes in the WRKY, NAC, and BZIP Transcription Factor Families Based on Single-Cell RNA Sequencing

To identify DEGs with cell type-specific patterns in the WRKY, NAC, and BZIP transcription factor families that may respond to abiotic stresses, we first obtained genes from the three transcription factor families. In total, 101, 135, and 113 genes of the WRKY, NAC, and BZIP transcription factor families were obtained from the National Center for Biotechnology Information (NCBI^2^) and previous studies (Nuruzzaman et al., 2010; Nan et al., 2020; Supplementary Table 1). The shared genes between *indica-japonica* DEGs and the WRKY, NAC, and BZIP transcription factor families in each cell type were visualized using the R package ggplot2 V3.2.1 (Wickham, 2016). The cell type-specific DEGs were visualized using the VlnPlot function in the R package Seurat V3.2.1 as noted above.

### Identification of Abiotic Stress-Induced Differentially Expressed Genes Based on Bulk RNA Sequencing

To further explore the different transcriptomic changes in cell types related to abiotic stresses between *indica* and *japonica* rice varieties, we integrated five bulk RNA sequencing datasets obtained under abiotic stresses, including cold (GSE145582; Rativa et al., 2020), drought (GSE92883; Muthurajan et al., 2018), and salinity (GSE109617, GSE58603, and GSE31874; Kumar et al., 2015; Zheng et al., 2019).

For cold stress (GSE145582), the comparison of cold-sensitive rice under control conditions with that under cold-stress conditions and the comparison of cold-sensitive rice under cold conditions with cold-tolerant rice under cold-stress conditions were used in this study. All treatments included two cDNA libraries and each library had four plant individuals as biological replications. Genes with a | logFC| > 2 and *P* < 0.05 were defined as cold DEGs. The R package Goplot V1.0.2 was used to visually overlap the *indica-japonica* DEGs and cold DEGs in the WRKY, NAC, and BZIP transcription factor families.

For drought stress (GSE92883), a comparison of drought-tolerant rice under control and drought-stress conditions was conducted in this study using GEO2R (Barrett et al., 2013). All treatments had two biological replications including six individual samples. The genes with a | logFC| > 2 and *P* < 0.05 were defined as drought DEGs. The R package biomaRt V2.40.5 (Durinck et al., 2009) was used for ID conversion of gene names. The R package Goplot V1.0.2 was used to visually overlap the *indica-japonica* DEGs and drought DEGs in the WRKY, NAC, and BZIP transcription factor families.

For salinity stress (GSE109617, GSE58603, and GSE31874), three comparisons were conducted in this study using GEO2R and the R package Limma V3.40.6 (Ritchie et al., 2015). The treatments in GSE109617 and GSE31874 had two biological replications, and the treatments in GSE58603 had three biological replications. Genes with a | logFC| > 2 and *P* < 0.05 were defined as salinity DEGs. The R package biomaRt V2.40.5 (Durinck et al., 2009) was used for ID conversion of gene names. The R package Pheatmap V1.0.12 (Kolde, 2019) was used to visually overlap the *indica-japonica* DEGs and salinity DEGs in the WRKY, NAC, and BZIP transcription factor families. The protein-protein interaction (PPI) network was analyzed using the STRING database^3^ and visualized by Cytoscape software (V3.7.0).

### Analysis of the Developmental Trajectory Based on Orthologous Markers in *Arabidopsis*

Orthologous analyses on the Ensembl Plant website^4^ were used to identify potential orthologous markers of QC cells in *Oryza sativa japonica* from *Arabidopsis.* Makers of QC cells in *Arabidopsis* were previously reported (Bruex et al., 2012; Ryu et al., 2019). The endodermal cells were subsets from both *indica* and *japonica* datasets and aligned to cell population clusters, as mentioned above. The counts of potential QC markers expressed in more than 3 cells were calculated to visualize the expression levels of QC markers in endodermis cells with the UMAP plot. Pseudotime trajectory analysis was performed using the R package Monocle V2.12.0 (Trapnell et al., 2014). The dynamic expression of potential QC markers along the pseudotime trajectory was visualized using the R package Pheatmap V1.0.12 and the plot_genes_in_pseudotime function in Monocle V2.12.0 (Trapnell et al., 2014).

## Results

### Cell Type-Specific Differentiation in Root Tips Between *Indica* and *Japonica* Rice Varieties

**FIGURE 1:**
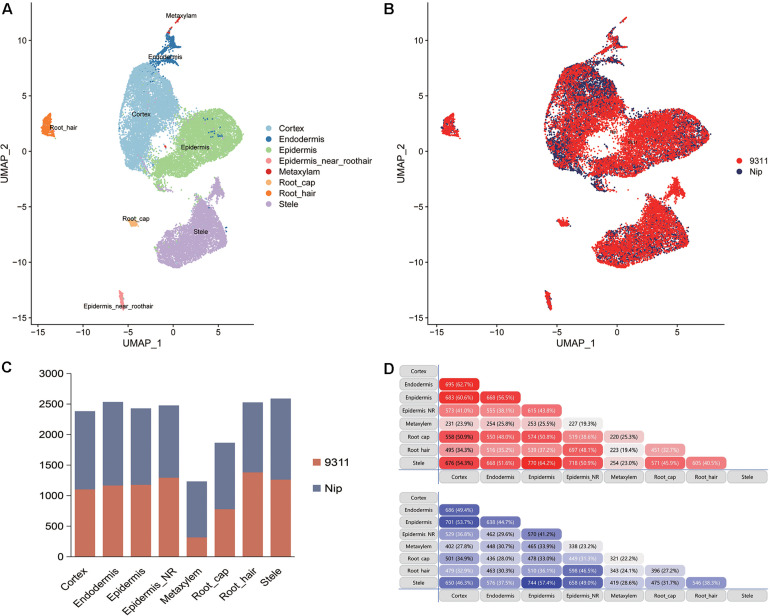
**(A)** UMAP plots showing the cell types from *Oryza sativa* ssp. *indica* variety 9311 and *Oryza sativa* ssp. *japonica* variety Nipponbare. **(B)** Cells from *Oryza sativa* ssp. *indica* variety 9311 and *Oryza sativa* ssp. *japonica* variety Nipponbare after removal of the batch effect. **(C)** Bar plot showing the number of differentially expressed genes (DEGs) between *indica* and *japonica* rice varieties in the eight cell types. Red bar, DEGs that were upregulated in *indica* variety 9311; blue bar, DEGs that were upregulated in *japonica* variety Nipponbare. **(D)** Shared DEGs in different cell types. Red bar, DEGs that were upregulated in *indica* variety 9311; blue bar, DEGs that were upregulated in *japonica* variety Nipponbare.

**FIGURE 2:**
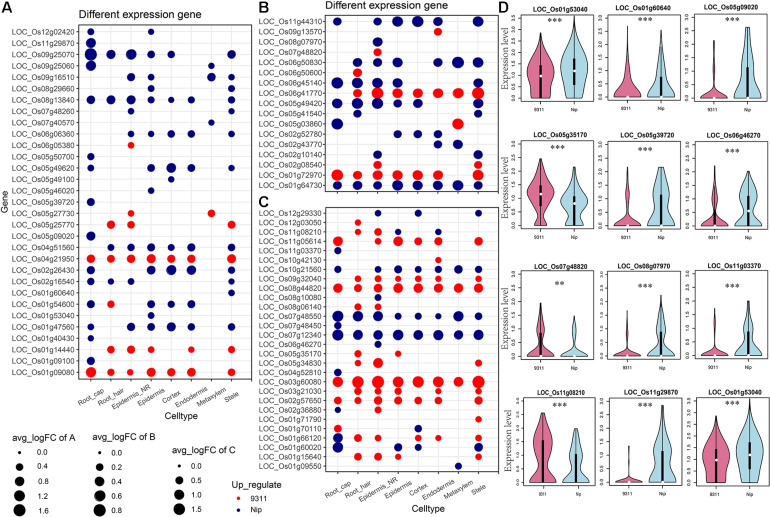
Differentially expressed genes (DEGs) between *indica* and *japonica* rice varieties in the WRKY **(A)**, BZIP **(B)**, and NAC **(C)** transcription factor families. **(D)** Violin plots showing the expression of differentially expressed genes of WRKY, BZIP, and NAC transcription factors between *indica* and *japonica* rice varieties in only one specific cell type. Red, *indica* rice variety 9311l; blue, *japonica* rice variety Nipponbare. The white point in each violin plot indicates the median of gene expression, and the error bars indicate variations of gene expression. ** indicates a *P* < 0.01; *** indicates a *P* < 0.001.

To explore the DEGs between *indica* and *japonica* rice varieties among cell types in root tips, we analyzed the single-cell RNA sequencing dataset from a published paper (GSE146035). Unsupervised clustering after UMAP with Seurat yielded eight major clusters in either *indica* or *japonica* rice varieties. Using markers in root tips, we defined eight main cell types, including the cortex, endodermis, epidermis, epidermis near the root hair, root hair, root cap, stele, and metaxylem, in both *indica* and *japonica* rice after removal of the batch effect (Figures 1A,B and Supplementary Figures 1, 2). To detect the expression patterns of genes between *indica* and *japonica* cultivated rice varieties in different cell types, we identified DEGs for each cell type (Supplementary Tables 2–9). Heterogenous gene expression between *indica* and *japonica* varieties was observed among the eight cell types. Although there was shared gene expression regulation, some DEGs between *indica* and *japonica* were observed in a cell type-specific manner (Figures 1C,D). In addition, to examine the function of cell type-specific DEGs, KEGG, and GO enrichments were conducted. The enrichments of KEGG and GO showed that the majority of DEGs were related to similar biological functions in the eight cell types (Supplementary Figures 6, 7), such as metabolic pathways and plant-pathogen interactions (*indica* variety 93–11 upregulated, Supplementary Figure 6A). However, some terms only enriched in a specific cell type (e.g., ether lipid metabolism and propanoate metabolism in the cortex), suggest the possibility that variations of differential functions between *indica* and *japonica* rice were in different cell types. Noticeably, some genes were enriched in the KEGG pathway of plant hormone signal transduction (Supplementary Figures 6, 7), which plays an important role in root development as well as stress responses. As the WRKY, NAC, and BZIP transcription factor families (Supplementary Table 1) have been demonstrated to play crucial roles in the rice abiotic stress response, we constructed a subset of DEGs between *indica* and *japonica* in the WRKY (Figure 2A), BZIP (Figure 2B), and NAC (Figure 2C) families that may be related to the adaptive evolution of cultivated *indica* and *japonica* rice varieties in different environments. The DEGs between *indica* and *japonica* in the WRKY, NAC, and BZIP families also showed a cell type-specific pattern, as shown in Figure 2D and Supplementary Figures 3, 4. For example, the cell type-specific DEGs in the root cap included *LOC_Os11g03370*, *LOC_Os05g39720*, *LOC_Os05g09020*, and *LOC_Os11g29870*; those in the epidermis near the root hair included *LOC_Os08g07970*, *LOC_Os07g48820, LOC_Os11g08210*, and *LOC_Os06g46270*; those in the root hair included *LOC*_*Os05g35170* and *LOC*_*Os06g50600*; *LOC*_*Os01g53040* was found in the epidermis; and *LOC_Os01g60640* was found in the stele (Figure 2D). These results indicate the existence of cell type-specific differentiation in root tips between *indica* and *japonica* varieties, which helps us elucidate the roles of different cell types in adaptive evolution in divergent environments.

### Transcriptomic Changes Related to Abiotic Stresses With Cell Type-Specific Patterns in Root Tips Between *Indica* and *Japonica* Rice

**FIGURE 3:**
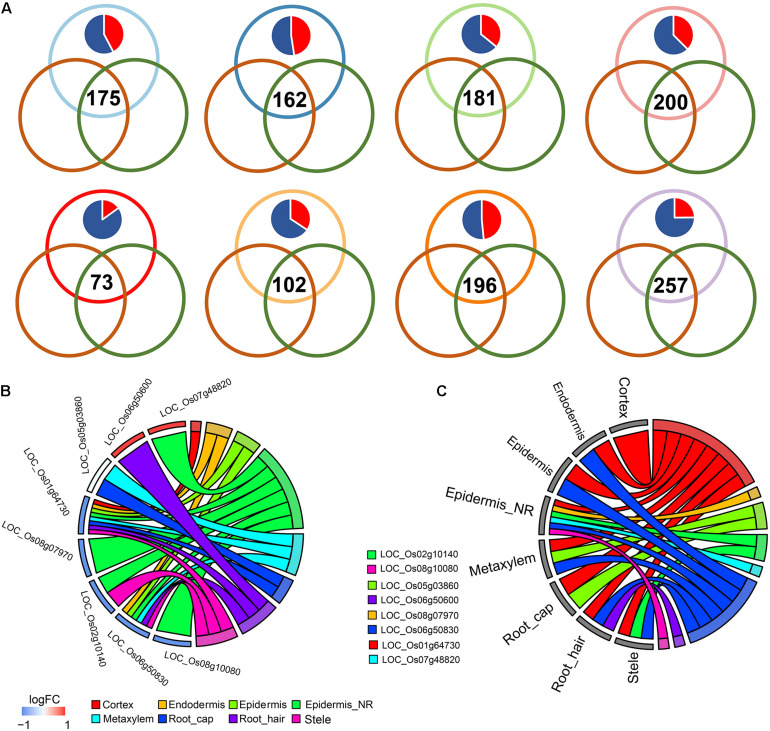
**(A)** Venn plots showing the differentially expressed genes (DEGs) shared during differentiation of *indica*-*japonica*, the comparison of cold-sensitive rice under control and cold-stress conditions and the comparison of cold-sensitive and cold-tolerant rice under cold-stress conditions in terms of the cell type in the cortex, endodermis, epidermis, epidermis near the root hair, metaxylem, root cap, root hair, and stele. Red in the pie chart indicates the percentage of DEGs that were upregulated in the *indica* variety 9311; blue in the pie chart indicates the percentage of DEGs that were upregulated in the *japonica* variety Nipponbare. **(B,C)** GOplot showing the *indica*-*japonica* DEGs that overlapped with two sets of cold-induced DEGs (belonging to the WRKY, BZIP, and NAC transcription factor families) in each cell type.

**FIGURE 4:**
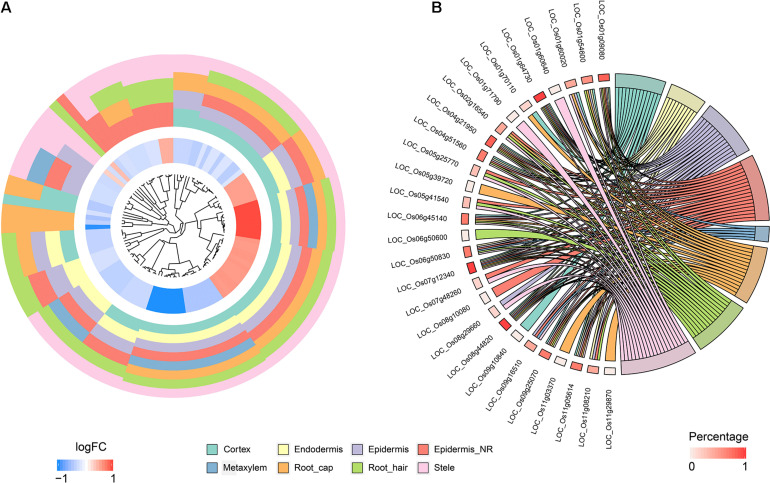
**(A)** The GOplot shows the *indica*-*japonica* DEGs that overlapped with drought-induced DEGs (belonging to the WRKY, BZIP, and NAC transcription factor families) in each cell type. The red gradient in **(B)** indicates the percentage of cell types showing differential gene expression between *indica* and *japonica* rice varieties.

**FIGURE 5:**
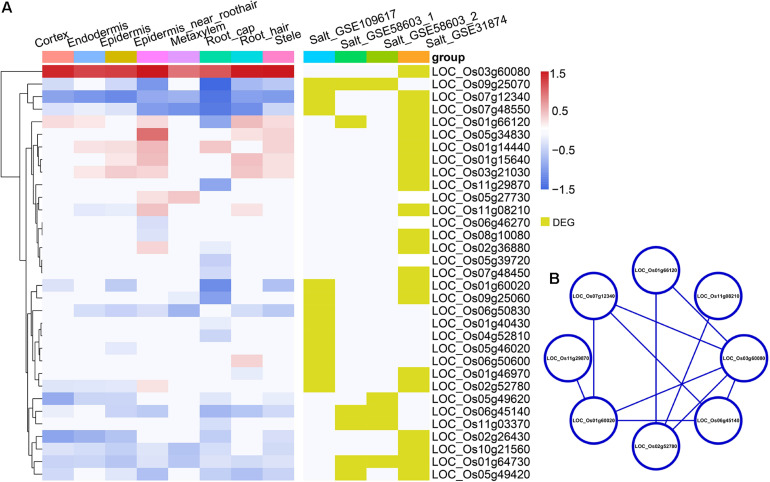
**(A)** The heatmap in the left panel shows the *indica*-*japonica* DEGs that overlapped with salinity-induced DEGs (belonging to the WRKY, BZIP, and NAC transcription factor families) in each cell type. The red gradient indicates the log FC of DEGs that were upregulated in the *indica* variety 9311; the blue gradient indicates the log FC of DEGs that were upregulated in the *japonica* variety Nipponbare. Yellow indicates DEGs in the corresponding dataset. **(B)** the protein-protein interaction analysis of *indica*-*japonica* DEGs that overlapped with salinity-induced DEGs (belonging to the WRKY, BZIP, and NAC transcription factor families).

To further explore the relationships between DEGs of *indica*-*japonica* and DEGs under abiotic stresses, we integrated some bulk RNA sequencing datasets under cold, drought and salinity treatments, in addition to their corresponding controls. We found that some DEGs between *indica* and *japonica* in the WRKY, NAC, and BZIP transcription factor families were also differentially expressed under cold, drought and salinity treatments compared to their controls (Figures 3–5). In this study, we defined the DEGs under cold, drought and salinity treatments in bulk RNA sequencing datasets as cold DEGs, drought DEGs and salinity DEGs.

Under cold treatment, cold DEGs (data from GSE145582) overlapped with *indica*-*japonica* DEGs in different cell types of rice root tips (Figure 3A); that is, some genes that showed differential expression during the differentiation of *indica* and *japonica* were also differentially expressed under cold treatment in specific cell types. Interestingly, the percentages of cold DEGs that were upregulated in the *japonica* rice variety were higher than those in the *indica* rice variety in all cell types in root tips (Figure 3A), which corresponded to the fact that the *japonica* rice varieties are more adapted to cold environments than the *indica* varieties. In addition, the eight cold DEGs were members of the WRKY, NAC, or BZIP transcription factor families (Figures 3B,C). Similar results were also found under drought and salinity treatments. Twenty-eight drought DEGs and 33 salinity DEGs overlapped with *indica*-*japonica* DEGs were also members of the WRKY, NAC, or BZIP transcription factor families (Figures 4, 5). The analysis of protein-protein interactions showed that eight salinity DEGs have indirect or direct interactions (Figure 5B). These results suggested that the cell type-specific DEGs between *indica* and *japonica* rice varieties have a relationship with adaptation to divergent environments.

### Analysis of Quiescent Center Cells Based on Orthologous Markers From *Arabidopsis*

**FIGURE 6:**
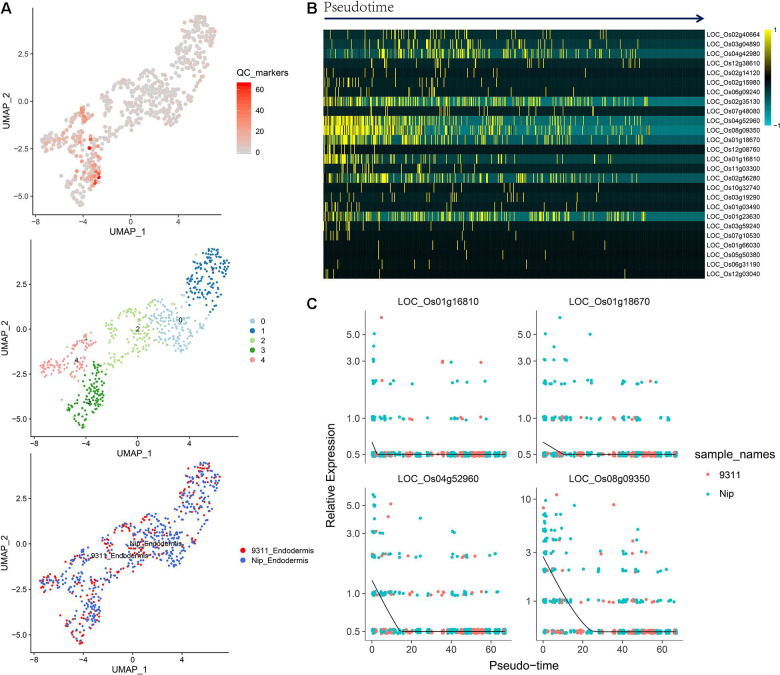
**(A)** UMAP plot of endodermal cells in rice roots showing the expression of 26 putative QC markers, subclusters and distribution of *indica* and *japonica* samples. **(B)** Heatmap showing the dynamic expression of 26 putative QC markers during the developmental trajectory based on single-cell pseudotime in endodermal cells. **(C)** Dynamic expression of 4 putative QC markers during the developmental trajectory based on single-cell pseudotime in endodermal cells.

To identify QC cells from single-cell RNA-Seq, we obtained orthologous markers (Supplementary Table 1) in rice from Ensembl Plants (see text footnote 4) using published QC markers from *Arabidopsis*. The cells in the endodermis cluster were used to conduct re-clustering and pseudotime analyses. Twenty-six rice orthologous markers that were expressed in at least 3 cell types were calculated and plotted for UMAP visualization. The 26 markers showed relatively high expression in subclusters 3 and 4 in both *indica* and *japonica* cells, and these cells may have a close relationship with stem cells in root tips (Figure 6A). In the pseudotime trajectory of endodermal cells, some genes showed relatively high expression in the initial stage (Figure 6B). For example, *LOC_Os01g16810*, *LOC_Os01g18670*, *LOC_Os04g52960*, and *LOC_Os08g09350* showed significant patterns of high expression upon initiation of the developmental trajectory (Figure 6C). These genes could help identify relatively small subpopulations of cells with relatively high expression in the initial developmental trajectory in roots, including stem cells and even QC cells.

## Discussion

Although the key roles of natural genetic variations in differentiation between the subspecies of *indica* and *japonica* rice varieties address disruption from environment changes and human preferences (Ma et al., 2015; Liu et al., 2019; Xu et al., 2020), differential gene expression also plays an important role in driving the adaptation of plants to divergent habitats (Liu et al., 2020; Wang et al., 2020). In this study, we identified eight cell types in rice root tips (Figures 1A,B) and compared the expression of genes between *indica* and *japonica* rice varieties in each cell type (Figures 1C,D) using a single-cell RNA sequencing dataset. We found variations in the total number of *indica*-*japonica* DEGs among cell types, for example, fewer DEGs were identified in the metaxylem cells than in other cell types (Figure 1C). In addition, the DEGs were different in eight cell types—for example, only 19.4% (*indica* variety 93-11, upregulated) and 24.1% (Nip variety, upregulated) of DEGs were shared by metaxylem cells and root hair cells (Figure 1D). In addition, some genes only showed differential expression in a single cell type, such as *LOC_Os01g53040* in the epidermis and *LOC_Os01g60640* in the stele (Figure 2D), which may not have been identified in traditional bulk RNA with mixed cell types. These differentially expressed genes with cell type-specific patterns suggested potential functional differences between *indica* and *japonica* rice in each cell type, as shown in the enrichments of KEGG and GO (Supplementary Figures 6, 7). In addition, some genes were enriched in the plant hormone signal transduction pathway (Supplementary Figures 6, 7), indicating that differentially expressed genes between *indica* and *japonica* rice were closely related to plant development and stress responses (Liu et al., 2020). These results suggested cell type-specific differentiation between *indica* and *japonica* rice varieties, which could help elucidate the roles of different cell types in the development and physiology of plants (Denyer et al., 2019; Ryu et al., 2019; Shulse et al., 2019; Liu et al., 2020).

As geographically and genetically diverged subspecies of Asian rice, *indica* and *japonica* rice varieties are divergent in their abilities to tolerate cold, drought and salinity stresses (Lu et al., 2009; Liu et al., 2018, 2020). Abiotic stress tolerance is a complex trait, and genes associated with abiotic stress may fall into two classes, including functional enzymes in biological processes and transcription factors that regulate gene expression (Huang et al., 2012; Liu et al., 2018). Both natural genetic variations and changed gene expression play an important role in abiotic stress tolerance in multiple species (Bian et al., 2020). For example, previous studies showed that SNPs or indels in the promoter or the coding sequence of genes (such as *COLD1*, *HAN1*, *bZIP73*, *LTG1*, *LTT7*, *qCTS-9*, *qPSR10*) can enhance the cold tolerance of *japonica* rice (Liu et al., 2013, 2019; Ma et al., 2015; Zhao et al., 2017; Xiao et al., 2018; Mao et al., 2019; Xu et al., 2020). A change in the promoter activity led to the modification of *HSFB2b* expression, which led to salt stress tolerance in soybeans (*Glycine* max) (Yolcu et al., 2020).

Previous studies have shown that these transcription factors play crucial roles in responding to diverse environmental stresses (Nijhawan et al., 2008; Zheng et al., 2009; Nuruzzaman et al., 2010; Liu et al., 2018; Nan et al., 2020). For example, *OsWRKY71* (WRKY gene) facilitates the adaptation of *japonica* rice to cold climates (Manu et al., 2017; Liu et al., 2018), and the overexpression of *ONAC045* (NAC gene) enhances rice drought and salt tolerance (Zheng et al., 2009). To further confirm the relationship between cell type-specific differentiation of *indica* and *japonica* rice varieties and various environments (different abiotic stresses), we also integrated a bulk RNA sequencing dataset obtained under cold, drought and salinity stress in this study and focused on *indica*-*japonica* DEGs in the BZIP, NAC, and WRKY transcription factor families (Figure 2). Our results showed a significant overlap between *indica*-*japonica* DEGs and stress-induced DEGs with cell type specificity in the root tips of cultivated rice (Figures 3–5). For example, we found that the *indica*-*japonica* DEGs *LOC*_*Os08g07970* (*OsbZIP64*, DEGs in the epidermis near the root hair upregulated in the *japonica* rice variety Nip), *LOC*_*Os02g10140* (*OsbZIP17*, DEGs in the epidermis near the root hair and stele upregulated in the *japonica* rice variety Nip) and *LOC_Os05g03860* (*OsbZIP38*, DEGs in the metaxylem and root cap) were also cold-induced DEGs in rice roots (Figures 3B,C). Among these genes, *LOC_Os08g07970* and *LOC_Os02g10140* were reported to have excessive linkage disequilibrium with the phenotype under cold stress, and their expression also responded to cold stress (Liu et al., 2018). *LOC_Os05g03860* dimerized with *OsOBF1* to mediate low-temperature signal switching in a previous study (Aguan et al., 1993; Shimizu et al., 2005a, b). Together, these results indicated *indica*-*japonica* differentiation in response to various environments with cell type-specific patterns in root tips, which helps reveal the roles of different cell types in the process of adaptation. In practical applications, DEGs in both *indica*-*japonica* rice varieties and under different stress treatments may provide some clues for breeding applications, as with the findings in previous study, which indicated that the selection of *COLD1*, *HAN1*, *bZIP73*, *LTG1*, *LTT7*, *qCTS-9*, and *qPSR10* can enhance the cold tolerance of *japonica* rice (Liu et al., 2013, 2019; Ma et al., 2015; Zhao et al., 2017; Xiao et al., 2018; Mao et al., 2019; Xu et al., 2020). The stress-induced DEGs in our study have potential as target genes for the improvement of stress tolerance in rice breeding applications.

The major benefit of single-cell RNA sequencing is the identification of cell types from many mixed cells in test samples, including rare cell types, such as QC cells. We calculated the expression of 26 orthologous markers from *Arabidopsis* QC cells (Ryu et al., 2019) in the UMAP plot (Figure 6A). The plots indicated relatively high expression in subcluster 3 and subcluster 4 (Figure 6A). The pseudotime analysis (Figures 6B,C) showed some genes (*LOC_Os01g16810*, *LOC_Os01g18670*, *LOC_Os04g52960*, and *LOC_Os08g09350*) with high expression in the initial stages of the developmental trajectory in roots. For example, the placement of *LOC_Os01g16810* with the GO term 1904961 (quiescent center organization) in the biological process category indicated its potential as a QC marker on the PLAZA website^5^. *LOC_Os01g18670*, *LOC_Os04g52960*, and *LOC_Os08g09350* were all related to the development of roots, as shown on the PLAZA website. In this study, although we cannot conclusively confirm QC cells from rice root tips, the genes highly expressed in the initial stage of root development (*LOC_Os01g16810*, *LOC_Os01g18670*, *LOC_Os04g52960*, and *LOC_Os08g09350*) could be potential markers for the identification of QC cells in the future.

In summary, based on analyses of single-cell RNA sequencing and bulk RNA sequencing datasets, we found some DEGs between *indica*-*japonica* rice varieties with cell type-specific patterns, and these genes were related to adaptative evolution in various environments during the process of *indica*-*japonica* differentiation. In addition, we found that *LOC*_*Os01g16810*, *LOC*_*Os01g18670, LOC_Os04g52960*, and *LOC_Os08g09350* may be potential markers of QC cells in rice root tips. These results provide new clues for understanding the development and physiology of plant roots during the process of adaptative divergence with cell type-specific patterns. In addition, these stress-induced DEGs provide potential target genes for the improvement of stress tolerance in rice, which are critical for genetic innovations in rice breeding applications that will contribute to food security in the future.

## Data Availability Statement

The original contributions presented in the study are included in the article/Supplementary Material, further inquiries can be directed to the corresponding author/s.

## Author Contributions

ChZ and ZL conceived and designed the study. ZW, DC, CF, and CoZ performed data analysis. All authors were involved in data interpretation and manuscript drafting, and approved the final manuscript.

## Conflict of Interest

The authors declare that the research was conducted in the absence of any commercial or financial relationships that could be construed as a potential conflict of interest.

## Footnotes

1 http://structuralbiology.cau.edu.cn/PlantGSEA/analysis.php

2 https://www.ncbi.nlm.nih.gov/

3 https://string-db.org/

4 https://plants.ensembl.org/index.html

5 https://bioinformatics.psb.ugent.be/plaza

## Supplementary Material

Supplementary Figure 1 Feature plots demonstrating the expression of the following selected genes on the UMAP plot in *Oryza sativa* ssp. *indica* variety 9311. Scaled expression levels are depicted using a red gradient (gray denotes lack of expression). Cortex marker genes: *LOC_Os12g02300*, *LOC_Os10g40510*, *LOC_Os04g46810*, *LOC_Os04g46820*, and *LOC_Os02g44310*; endodermis marker genes: *LOC_Os01g48770*, *LOC_Os05g39960*, *LOC_Os01g16890*, *LOC_Os04g34630*, and *LOC_Os12g38689*; epidermis marker genes: *LOC_Os11g06720*; epidermis near root hair marker genes: *LOC*_*Os02g15700*, *LOC*_*Os07g43670*, *LOC*_*Os05g04450*, *LOC*_*Os03g12290*, and *LOC_Os03g61470*; metaxylem marker genes: *LOC_Os01g15610*, *LOC_Os07g01370*, *LOC_Os03g63390*, *LOC_Os01g73980*, and *LOC_Os07g44450*; root cap marker genes: *LOC_Os04g46630*, *LOC_Os05g47939*, *LOC_Os01g14540*, *LOC_Os01g73729*, and *LOC_Os01g73700*; root hair marker genes: *LOC_Os10g39160*, *LOC_Os10g39890*, *LOC_Os07g31610*, *LOC_Os01g07060*, and *LOC_Os07g35860*; stele marker genes: *LOC_Os02g43660*, *LOC_Os07g07860*, *LOC_Os07g07790*, *LOC_Os01g15830*, and *LOC_Os06g46799*.

Supplementary Figure 2 Feature plots demonstrating the expression of the following selected genes on the UMAP plot in *Oryza sativa* ssp. *japonica* variety Nipponbare. Scaled expression levels are depicted using a red gradient (gray denotes lack of expression). Cortex marker genes: *LOC*_*Os10g40520*, *LOC*_*Os10g40510*, *LOC*_*Os12g36210*, *LOC*_*Os03g01300*, and *LOC*_*Os04g46810*; endodermis marker genes: *LOC_Os03g13170*, *LOC_Os07g33997*, *LOC_Os01g16890*, *LOC_Os04g34630*, and *LOC_Os05g39960*; epidermis marker genes: *LOC_Os05g04500*; epidermis near root hair marker genes: *LOC_Os06g20150*, *LOC_Os03g25280*, *LOC_Os03g25300*, *LOC_Os03g61470*, and *LOC_Os03g12290*; metaxylem marker genes: *LOC_Os01g15610*, *LOC_Os07g01370*, *LOC_Os03g63390*, *LOC_Os01g73980*, and *LOC_Os05g01810*; root cap marker genes: *LOC*_*Os06g51050*, *LOC*_*Os10g31660*, *LOC*_*Os04g46630*, *LOC*_*Os01g73700*, and *LOC*_*Os05g15690*; root hair marker genes: *LOC_Os02g44470*, *LOC_Os01g18970*, *LOC_Os10g11730*, *LOC_Os07g35860*, and *LOC_Os06g22919*; stele marker genes: *LOC_Os02g43660*, *LOC_Os07g07860*, *LOC_Os07g07790*, *LOC_Os01g15830*, and *LOC_Os06g46799.*

Supplementary Figure 3 Violin plots showing the expression of differentially expression genes of BZIP transcription factors between *indica* and *japonica* rice varieties in eight cell types. Red, *indica* rice variety 9311; blue, *japonica* rice variety Nipponbare.

Supplementary Figure 4 Violin plots showing the expression of differentially expression genes of NAC transcription factors between *indica* and *japonica* rice varieties in eight cell types. Red, *indica* rice variety 9311; blue, *japonica* rice variety Nipponbare.

Supplementary Figure 5 Violin plots showing the expression of differentially expression genes of WRKY transcription factors between *indica* and *japonica* rice varieties in eight cell types. Red, *indica* rice variety 9311; blue, *japonica* rice variety Nipponbare.

Supplementary Figure 6 Top15 KEGG pathways **(A)**, GO biological processes **(B)**, GO cellular components **(C)** and GO molecular functions **(D)** of DEGs (upregulated in *indica* rice variety 9311) between *indica* and *japonica* rice in eight cell types, respectively.

Supplementary Figure 7 Top15 KEGG pathways **(A)**, GO biological processes **(B)**, GO cellular components **(C)** and GO molecular functions **(D)** of DEGs (upregulated in *japonica* rice variety Nipponbare) between *indica* and *japonica* rice in eight cell types, respectively.
